# Evaluation of HemogloBind™ treatment for preparation of samples for cholinesterase analysis

**DOI:** 10.4236/abb.2013.412136

**Published:** 2013-12-01

**Authors:** Kevin G. McGarry, Ryan A. Bartlett, Nicholas J. Machesky, Thomas H. Snider, Robert A. Moyer, David T. Yeung, Matthew K. Brittain

**Affiliations:** 1 Battelle Biomedical Research Center, Battelle Memorial Institute, Columbus, USA; 2 National Institutes of Health/National Institute of Neurological Disorders and Stroke, Bethesda, USA

**Keywords:** Acetylcholinesterase, Cholinesterase, HemogloBind™, Sample Preparation, Hemoglobin

## Abstract

Acetylcholine is an essential neurotransmitter found throughout the nervous system. Its action on postsynaptic receptors is regulated through hydrolysis by various carboxylesterases, especially cholinesterases (ChEs). The acute toxicity of organophosphate (OP) compounds is directly linked to their action as inhibitors of ChE. One widely used assay for evaluating ChE activity is a spectrophotometric method developed by Ellman *et al.* When the enzyme source is from tissues or, in particular, blood, hemoglobin displays a spectrophotometric peak at the same wavelength used to analyze cholinergic activity. This creates a substantial background that interferes with the Ellman’s assay and must be overcome in order to accurately monitor cholinesterase activity. Herein, we directly compare blood processing methods: classical method (1.67 ± 0.30 U/mL) and HemogloBind™ treatment (1.51 ± 0.17 U/mL), and clearly demonstrate that pretreatment of blood samples with Hemoglobind™ is both a sufficient and rapid sample preparation method for the assessment of ChE activity using the Ellman’s method.

## 1. INTRODUCTION

Acetylcholine is an essential neurotransmitter found throughout both the central nervous system (CNS) and peripheral nervous system (PNS). The actions of acetylcholine on its receptors are normally terminated when it is hydrolyzed by various carboxylesterases, most notably acetylcholinesterase (AChE) and butyrylcholinesterase (BChE, pseudocholinesterase) [1]. Multiple isoforms of AChE are found throughout the body at neuromuscular junctions, cholinergic synapses in the brain, and in red blood cell (RBC) membranes, while BChE is found primarily in the plasma [1].

**Figure 1:**
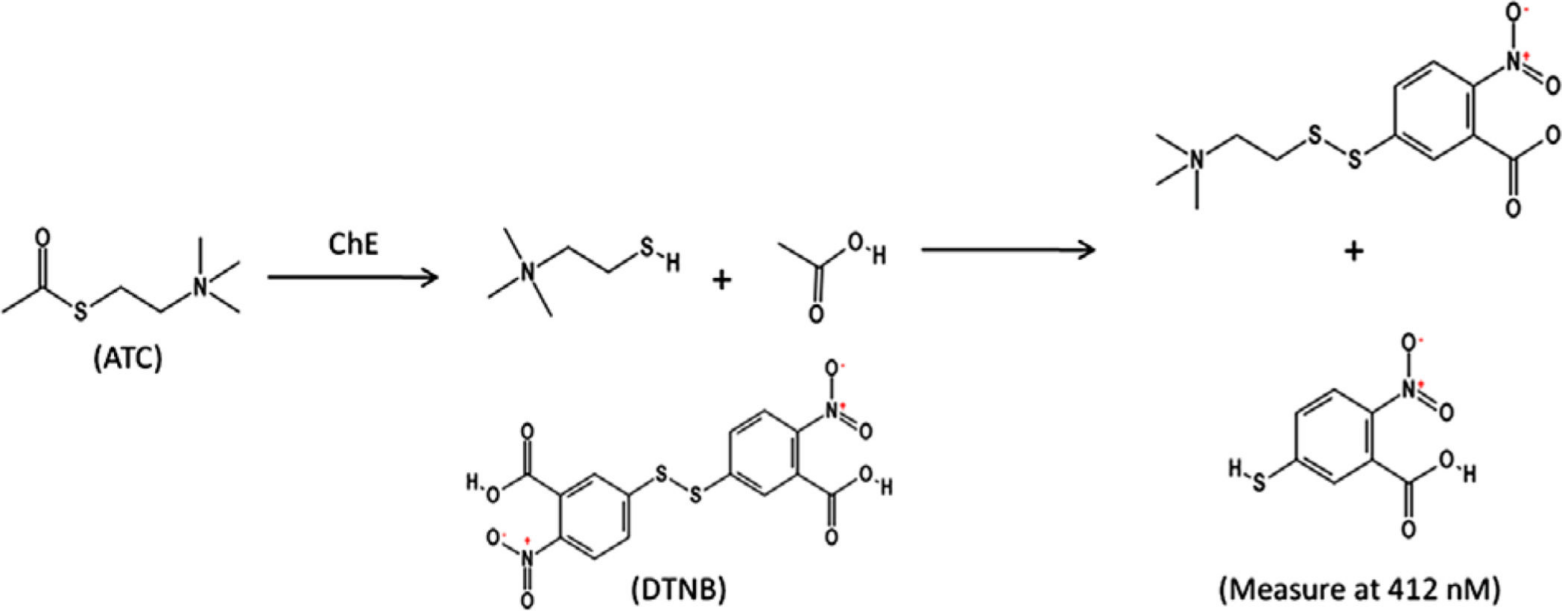
Ellman’s reaction.

In 1961, Ellman *et al.* developed a spectrophotometric method which is widely used to evaluate both AChE and BChE activity [2]. *In vivo*, AChE hydrolyzes acetylcholine producing an acetate and a choline molecule. The Ellman’s assay uses acetylthiocholine (ATC), a nonnative substrate, for the measurement of AChE activity *in vitro*. BChE and other non-specific carboxylesterases found in the plasma also hydrolyze ATC. The activity in the plasma when added to RBC membrane activity represents total ChE activity. The hydrolysis reaction results in the formation of a free thiol. The thiol reacts with Ellman’s reagent (5,5’-dithiobis-(2-nitrobenzoic acid) or DTNB), cleaving the disulfide bond. The product of this rapid chemical reaction is the yellow colored, 2-nitro-5-thiobenzoate (NTB) [2]. The concentration and formation of NTB can be determined kinetically using a spectrophotometer at a wavelength of 412 nM. The rate at which NTB is formed can be directly linked to enzymatic hydrolysis activity and is the basis of the Ellman’s reaction shown below (Figure 1).

Hemoglobin displays a spectrophotometric peak that significantly overlaps with the spectrophotometric peak of NTB. This creates a substantial background that interferes with the Ellman’s assay and must be overcome in order to accurately monitor cholinesterase activity in blood samples. For years, researchers have recognized the spectral overlap between hemoglobin and the NTB reaction product and have worked to overcome it. There are several methods, such as the RBC membrane preparation and the dilutional approach accepted for the handling of samples prior to analysis using the Ellman’s method [3]. For samples that cannot be diluted due to low ChE activity, and/or where the use of a native substrate is necessary, a highly effective radiometric method for acetylcholine hydrolysis has been developed [4]. The obvious pitfall to this method is the need to work with, and dispose of, radioactive material.

The classical method for removing hemoglobin from a sample is the RBC membrane preparation approach which involves separating plasma from red blood cells (RBCs) by centrifugation, lysing the RBCs, and washing the cell membranes to remove hemoglobin [3]. Unfortunately, there are two key limitations of this approach. First, when a large number of samples are to be analyzed, such as following the Tokyo subway attack, preparing membranes prior to analysis is labor intensive and slow. Second, this approach removes plasma containing BChE and the read-through isoform of AChE, the latter of which is expressed at elevated levels as a stress response [5–7]. Therefore, total ChE activity cannot be assessed, as this method retains only the membrane associated ChEs while excluding the soluble ChEs. If total ChE activity is desired, then an additional analysis must be performed to assess the soluble fraction found in the plasma. Then, the results of the two assays are added together to give total ChE activity.

An alternative method for total ChE analysis of whole blood was developed that directly addresses the spectral overlap issue by removing hemoglobin from test samples. A commercially available bead-based product, Hemoglo-Bind™, (Biotech Support Group: HO145), is designed to remove hemoglobin from serum and plasma while allowing the supernatant to retain its enzymatic and biological activity [8]. After binding hemoglobin, the beads are removed by a brief, low speed centrifugation. Once hemoglobin is removed, a standard Ellman’s method can be used for analysis of total ChE. Here we show that the use of HemogloBind™ to remove hemoglobin from whole blood samples (HemogloBind™ method) is a cost effective, high-throughput sample preparation alternative for obtaining total ChE activity and comparable to the classical method.

## 2. MATERIALS AND METHODS

A comparison between the HemogloBind™ method and the widely accepted classical (RBC membrane preparation) approach for reducing or removing hemoglobin was performed using whole blood samples obtained from male Hartley guinea pigs. The methods were compared in terms of reducing and/or eliminating the background caused by the presence of hemoglobin in test samples while maintaining ChE activity.

### 2.1. RBC Membrane and Plasma Preparations (Classical Method)

To ensure complete lysis, whole blood samples were freeze fractured and subsequently diluted in Millipore water at a ratio of 1:10. RBC membranes were pelleted by centrifugation at 20,000 × g for 15 minutes. After removal of the supernatant, the pellet was resuspended in 1 mL of 1× PBS (Sigma Aldrich: P3813; 0.01 M phosphate buffered saline (NaCl 0.138 M, KCl 0.0027 M)) to wash the membranes. Centrifugation and resuspension were repeated until hemoglobin was sufficiently reduced for the sample. At this point, the pellet was resuspended in its original volume with 1× PBS and immediately analyzed for ChE activity [3]. In order to obtain total ChE activity, the plasma also was analyzed. To separate plasma, whole blood was centrifuged at 1300 × g for 10 minutes at 4°C. After centrifuging, the plasma was removed for ChE activity analysis.

### 2.2. HemogloBind™ Treatment

Whole blood was first frozen to freeze fracture RBC membranes and, upon thawing, a 1:10 dilution in Millipore water was made to ensure complete lysis of the RBCs. HemogloBind™, a poly-electrolyte that has a high affinity for hemoglobin, was added at a 1:1 ratio to the diluted blood. The sample was vortexed for 30 seconds, and then placed on a rocker for 15 – 20 minutes to mix thoroughly. The sample was then centrifuged for 2 minutes at ~8000 × g to pellet the HemogloBind™ beads [8]. The supernatant containing the sample of interest, depleted of hemoglobin, was retained and immediately analyzed for ChE activity. During data analysis, a dilution factor of 1.75 was applied to account for removal of the solid matrix after HemogloBind™ treatment of samples.

Samples for all preparation methods were analyzed for ChE activity immediately upon completion of the sample preparation. However, prior results in our laboratory have shown that ChE activity is unaffected by storing the prepared samples at ≤−70°C and analyzing them at a later time (data not shown).

### 2.3. Cholinesterase Activity Analysis of Samples

Following preparation by one of the two methods described above, sample analysis was performed in a manner similar to that described by Ellman *et al.* [2]. Using a 96-well optical bottom plate (Fisher Scientific: 12-566-73) 100 µL of test sample was added to 60 µL PBS, pH 7.5, in triplicate. Ellman’s Reagent (DTNB; 5,5’-dithiobis(2-nitrobenzoic acid) Sigma Aldrich: D8130) was added to a final concentration of 500 µM. Acetylthiocholine iodide (ATC) (Sigma Aldrich: A5751) was used as the reaction substrate and added to a final concentration of 1 mM immediately prior to analysis. Upon addition of the substrate, the plate was transferred to a Spectramax Plus384™ (Molecular Devices) plate reader and kinetic data were obtained at 412 nm every 15 seconds for a period of 10 minutes. Using Beer’s Law (A = ELC), where E is the extinction coefficient of DTNB (13,600 M^−1^·cm^−1^) and L is 1 cm path length, the enzyme activity in µmoles·min^−1^·mL^−1^ (U/mL) for each sample was calculated.

### 2.4. Statistics

A mixed effects analysis of variance (ANOVA) model was used to compare ChE results from different sample preparation methods after accounting for repeated measures by multiple test operators on each animal. The model was fit using SAS^®^ version 9.3 MIXED procedure.

## 3. RESULTS AND DISCUSSION

The RBC membrane preparation method is the classical approach for removing hemoglobin from whole blood prior to AChE analysis. Using this method, five whole blood samples taken from five male Hartley guinea pigs were processed for analysis (Table 1). The mean ± standard deviation cholinesterase activity using the RBC membrane preparation was determined to be 0.90 ± 0.21 U/mL. This traditional blood preparation approach is a low capacity, highly reproducible, trusted method. However, RBC membrane preparation is relatively labor-intensive and not optimal for high throughput screening. In addition, plasma analysis is necessary to obtain total ChE activity which requires additional time and effort. The mean plasma ChE activity was 0.77 ± 0.21 U/mL, so the mean total blood ChE activity was 1.67 ± 0.30 U/mL.

An alternative method for sample preparation was desired without compromising the integrity and reproducibility of the sample activity. Thus, a comparison was made using HemogloBind™ treatment prior to analysis using the standard Ellman’s approach. The results following HemogloBind™ treatment of whole blood from the same set of five male guinea pigs showed that total cholinesterase activity was 1.51 ± 0.17 U/mL (Table 1). In comparison, the sum of RBC membrane preparation ChE activity and plasma ChE activity was 1.67 ± 0.30 U/mL. Using a mixed effects ANOVA, it was determined that there was no significant statistical difference between the mean ChE activity of samples prepared using HemogloBind™ and classical approaches (p = 0.085).

**Figure 2:**
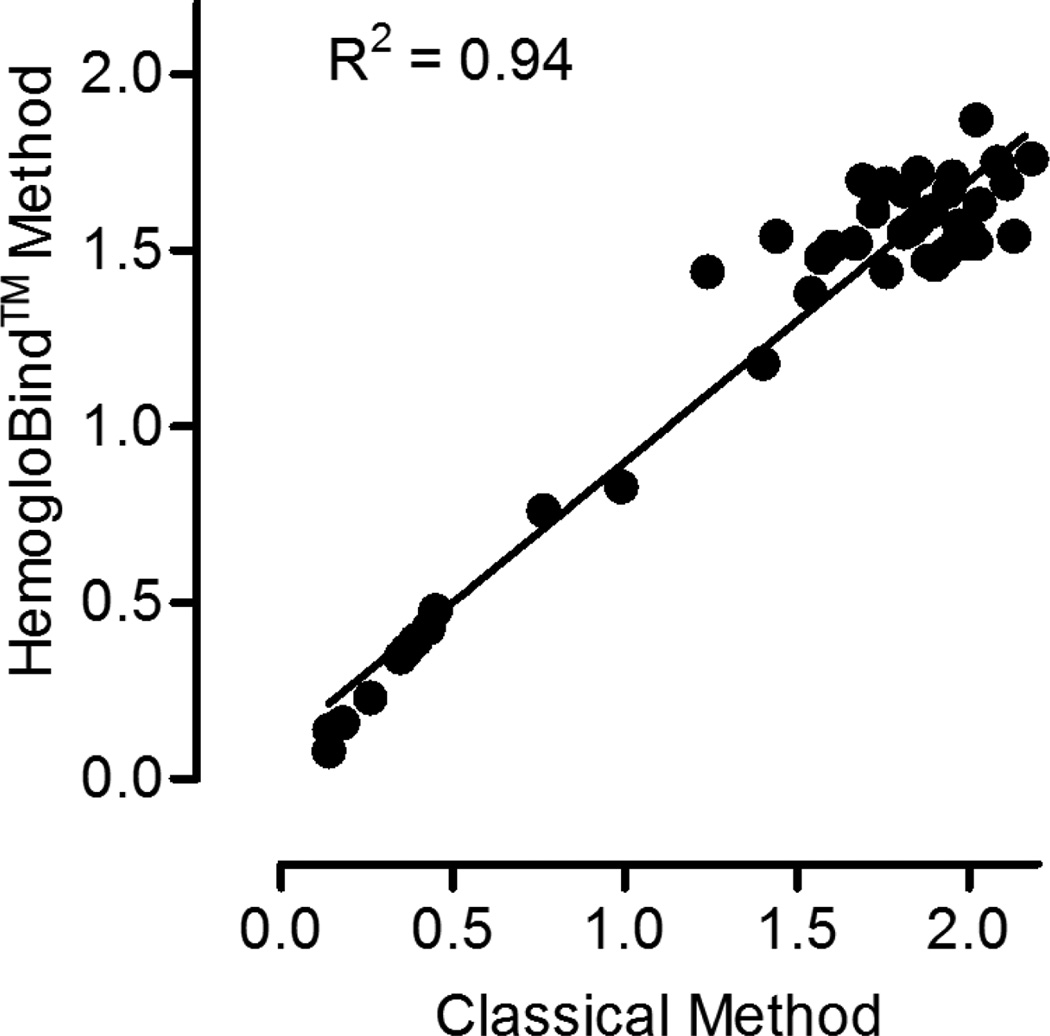
Cholinesterase activity comparison. Cholinesterase activity values determined from HemogloBindTM vs classical blood (RBC + plasma) processing methods (U/mL). n = 45. Slope = 0.80; 95% CI = 0.73, 0.86; p < 0.0001.

In order to provide additional comparisons between the classical blood preparation method and the Hemoglo-Bind™ method among a broad range of ChE activity, the two methods were compared using blood from guinea pigs challenged with cholinesterase inhibitors (Figure 2). These data (n = 45) show that both methods yield comparable results. The linear correlation slope is 0.80 with a 95% confidence interval of 0.73, 0.86; p < 0.0001. A slope less than 1 indicates that the HemogloBind™ method consistently results in lower ChE activity values relative to the classical method. This trend was also observed in Table 1, but was not statistically significant, most likely due to the smaller sample size (n = 5). Ultimately, this difference between the two methods is consistent and reproducible as indicated by the R^2^ value of 0.94. These data suggest that each sample preparation method is valid for evaluating ChE activity.

Overall, the HemogloBind™ preparation method has the potential for use as a high capacity method for blood processing prior to determining total ChE activity. The largest advantage is the single sample preparation, rather than the addition of both RBC membrane and plasma-preparations, as needed when using the classical method.

Also, the RBC membrane preparation is fairly labor intensive, whereas the HemogloBind™ treatment is a single incubation and a short, low speed centrifugation. Both of these advantages allow for a large number of hemolyzed samples to be prepared for analysis quickly.

## 4. CONCLUSION

In conclusion, the HemogloBind™ preparation method provides an alternative, high throughput approach for sample handling prior to enzyme activity analysis while retaining total cholinesterase activity and directly addressing the issue of spectral overlap through the removal of hemoglobin from test samples. The resulting total ChE activity was comparable to the traditional preparation technique while reducing labor and overall cost. High throughput techniques are needed to allow processing of large numbers of samples quickly for research purposes and potential mass screening during a large scale, acute poisoning situation, and the HemogloBind™ treatment method accomplishes this.

## Figures and Tables

**Table 1:** Total cholinesterase activity: classical approach vs HemogloBind™ treatment in the male Hartley guinea pig.

	RBC Membranes (classical)	Plasma (classical)	Total ChE Activity (RBC + Plasma)	HemogloBind™ Treatment
Mean (U/mL)	0.90	0.77	1.67	1.51
Std. Dev.	0.21	0.21	0.30	0.17

Note: Mean values represent the average of three technical replicate values from five male Hartley guinea pig samples analyzed by two operators.
